# Dual‐Responsive MXene‐Functionalized Wool Yarn Artificial Muscles

**DOI:** 10.1002/advs.202402196

**Published:** 2024-04-22

**Authors:** Liuxiang Zhan, Shaohua Chen, Yangyang Xin, Jian Lv, Hongbo Fu, Dace Gao, Feng Jiang, Xinran Zhou, Ni Wang, Pooi See Lee

**Affiliations:** ^1^ Shanghai Frontier Science Research Center for Advanced Textiles College of Textiles Donghua University Shanghai 201620 China; ^2^ Engineering Research Center of Technical Textile Ministry of Education College of Textiles Donghua University Shanghai 201620 China; ^3^ School of Materials Science and Engineering Nanyang Technological University 50 Nanyang Avenue Singapore 639798 Singapore

**Keywords:** artificial muscles, MXene, photothermal actuators, smart textile, wool

## Abstract

Fiber‐based artificial muscles are promising for smart textiles capable of sensing, interacting, and adapting to environmental stimuli. However, the application of current artificial muscle‐based textiles in wearable and engineering fields has largely remained a constraint due to the limited deformation, restrictive stimulation, and uncomfortable. Here, dual‐responsive yarn muscles with high contractile actuation force are fabricated by incorporating a very small fraction (<1 wt.%) of Ti_3_C_2_T_x_ MXene/cellulose nanofibers (CNF) composites into self‐plied and twisted wool yarns. They can lift and lower a load exceeding 3400 times their own weight when stimulated by moisture and photothermal. Furthermore, the yarn muscles are coiled homochirally or heterochirally to produce spring‐like muscles, which generated over 550% elongation or 83% contraction under the photothermal stimulation. The actuation mechanism, involving photothermal/moisture‐mechanical energy conversion, is clarified by a combination of experiments and finite element simulations. Specifically, MXene/CNF composites serve as both photothermal and hygroscopic agents to accelerate water evaporation under near‐infrared (NIR) light and moisture absorption from ambient air. Due to their low‐cost facile fabrication, large scalable dimensions, and robust strength coupled with dual responsiveness, these soft actuators are attractive for intelligent textiles and devices such as self‐adaptive textiles, soft robotics, and wearable information encryption.

## Graphical abstract

This work reports high‐performance yarn‐based artificial muscles with dual responses by introducing Ti_3_C_2_T_x_ MXene/cellulose nanofibers composites into twisted and coiled wool yarns. Due to their low‐cost facile fabrication, large scalable dimensions, and robust strength couple with dual responsiveness, these soft actuators offer new opportunities for broad applications in the fields of soft robotics, smart textiles, and intelligent systems.

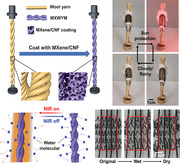

## 1 Introduction

Smart textiles have been rapidly developed to demonstrate the capabilities of sensing, actuating, communicating, memorizing, learning, energy harvesting, temperature, moisture regulation, etc.^[^ ^1^ ^]^ Since the global market of smart textiles is estimated to have an enormous worth of $160 billion by 2026,^[^ ^2^ ^]^ the exciting potential for innovations and development of smart textiles is clearly visible. In particular, smart stimulus‐responsive functions are of special interest to be implemented on fibers and fabrics for changing the morphology and porosity of textiles, for example, to increase comfort via better moisture and thermal management.^[^ ^3^ ^]^ As basic units of textiles, fibers, and yarns can be processed into artificial muscles (actuators) by inserting twists, which could be further fabricated through well‐established textile technologies into versatile fabric actuators with 1D, 2D, or 3D deformations.^[^ ^4^ ^]^ These fiber/yarn artificial muscles can be applied not only in smart clothing and wearables to realize numerous advanced functions, but also in engineering fields such as soft robotics and human‐robot interfaces.^[^ ^5^ ^]^

Various types of twisted fiber artificial muscles have been developed,^[^ ^6^ ^]^ which can generate external work and produce deformations under numerous stimulation conditions, including heat, humidity, pH, pressure, electric current, light, etc.^[^ ^4^, ^7^ ^]^ Among them, light‐driven fiber/yarn actuators, which are based on the energy conversion from light to actuation, have attracted particular interest of researchers due to their advantages of remote and accurate control and flexible adjustability.^[^ ^8^ ^]^ Light‐driven actuation is mainly ascribed to the volume changes induced by the photothermal effect (thermal expansion/shrinkage due to the change of bond length, polymer conformation, or moisture content) or photochemical effect.^[^ ^9^ ^]^ A few light‐driven fiber‐based artificial muscles have been reported by using photothermal materials such as graphene oxide (GO),^[^ ^10^ ^]^ carbon nanotube (CNT),^[^ ^11^ ^]^ and liquid crystal elastomer (LCE).^[^ ^12^ ^]^ However, complex processes, limited deformation, and poor wearability (e.g., unable to manage human body heat and moisture) restrict their application potentials for the fabrication of smart textiles and subsequent commercialization. It is highly desirable to develop artificial muscles that can respond to photothermal and moisture stimuli, generate reversible large actuation stress/stroke with robust stability, and can be well integrated into smart textiles.

**Figure 1:**
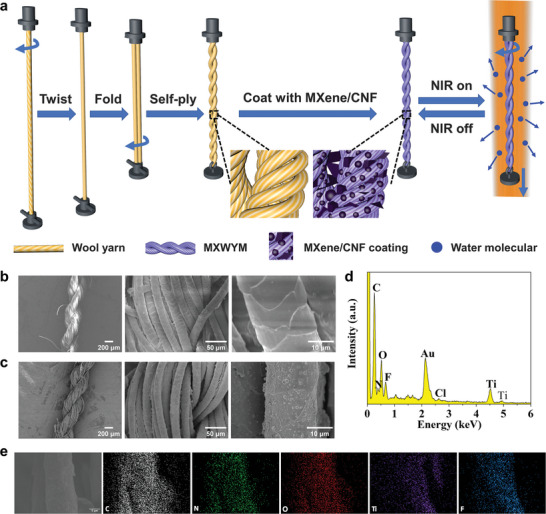
Fabrication and morphology of self‐plied MCWYMs and the elemental analysis. a) Schematic illustration of the fabrication of a two‐ply, torque‐balanced MCWYM. b,c) SEM images of (b) a neat WYM and (c) a MCWYM. d) EDX spectrum and e) mapping of the MCWYM.

Selecting suitable textile materials, integrating with novel responsive materials, and rationally designing the configuration are key strategies for developing high‐performance artificial muscles for smart textiles. Thus, natural fibers are promising options due to their low cost, wearing comfort, and ease of processing.^[^ ^13^ ^]^ Among these, wool is an interesting candidate for twist‐yarn‐based artificial muscles because of the excellent wearing comfort,^[^ ^14^ ^]^ biocompatibility to the human body, and especially, the gigantic hygroscopic expansion.^[^ ^15^ ^]^ Ti_3_C_2_T_x_ MXene, one of the 2D metal carbides,^[^ ^16^ ^]^ could be introduced into wool yarn muscles (WYMs) as a highly efficient photothermal agent (**Figure** **1**a) due to its outstanding photothermal conversion efficiency and broad light absorption in the visible to near‐infrared (NIR) range.^[^ ^17^ ^]^ In this work, dual (moisture and photothermal) responsive artificial muscles based on MXene/cellulose nanofiber functionalized WYMs (MCWYMs) are developed to realize different types of actuations such as contraction and elongation. Two working modes for generating high contractile stress and large stroke are also demonstrated respectively by using self‐plied and coiled MCWYMs. The direct strain measurements, simultaneous monitoring of water loss and temperature change, and finite element simulation are employed to reveal the photo‐thermo‐mechanical transducing behavior and hence the actuation mechanism of MCWYMs. Finally, potential applications of these artificial muscles were demonstrated in the fields of soft robotics, smart switches, intelligent textiles with adaptive moisture/thermal management, and information encryption.

## 2 Results and Discussion

### 2.1 Fabrication of Self‐Plied MCWYMs

Wool is a keratin‐based animal fiber that contains a variety of proteins and polypeptides. The hydrophilic amino acid residues in keratin and the porous structure of the wool fiber aggregates contribute to the hygroscopic properties of wool yarn.^[^ ^18^ ^]^ Moreover, wool fiber shows an obvious hygroscopic expansion from dry to wet, that is, 16% expansion in the radial direction and over 40% expansion in the volume.^[^ ^15^ ^]^ Thus, wool was chosen as an ideal material for fabricating artificial muscles in this work. The fabrication process of a neat wool yarn muscle (WYM) was schematically illustrated in Figure 1a. To significantly enhance the actuation of a WYM during the reversible water absorption and desorption process, twists were inserted into the yarn followed by folding and self‐plying to form a torque‐balanced structure, which also eliminated the tendency to untwist (Figure S1, Supporting Information).^[^ ^7^, ^19^ ^]^ Note that the twist direction of self‐plying was opposite to that of individual fibers because the initially twisted fibers were partially relaxed to provide some twists for self‐plying, resulting in a torque‐balanced WYM. For example, if the individual fiber had an S (left‐handed) twist, the self‐plied WYM would have a Z (right‐handed) twist.

Compared with other 2D nanomaterials such as graphene oxide (GO), Ti_3_C_2_T_x_ MXene has a superior light‐absorption ability^[^ ^17^, ^20^ ^]^ and was selected as the photothermal agent for WYMs to endow them with light‐response capability. Previous studies showed that biocompatible cellulose nanofibers (CNF) can tightly bind MXene nanosheets through hydrogen bonds to form MXene/CNF composites with improved moisture response rate and water absorption.^[^ ^21^ ^]^ Therefore, a MXene/CNF composite (Figure S1, Supporting Information) was coated on the WYMs to enhance both the water absorption and photothermal response, leading to dual‐response MCWYMs (Figure 1a; Figure S2, Supporting Information). SEM images (Figure 1b,c) show that MXene sheets were continuously coated on the surface of wool fibers, as evidenced by the blurred cuticles after coating. Some flakes were also deposited in the gaps between fibers and a few round particle aggregates presented on the surface of wool fibers. These aggregates could be attributed to the strong hydrogen bonding interaction between the oxygen‐containing groups of CNF and the surface functional groups of MXene in the high‐concentration dispersion.^[^ ^22^ ^]^ Meanwhile, these groups of MXene and CNF can also lead to their adsorption on the wool fiber with many peptide bonds. For example, Figure S2a–c (Supporting Information) shows that the MXene/CNF mixture was more likely to stick to the smooth walls of the glass bottle than neat MXene does. The strong hydrogen bonding interaction with wool fibers could be the main factor for their successful coating on wool fibers,^[^ ^23^ ^]^ which was confirmed by the results of Fourier‐transform infrared spectroscopy (Figure S3a, Supporting Information, no new peaks were observed). The presence of MXene on the coated wool was also confirmed by the ultraviolet‐visible spectroscopy (Figure S3b, Supporting Information). The addition of MXene increased the light absorbance of wool in both the visible and NIR range, showing an absorption peak at 780 nm.^[^ ^24^ ^]^ In addition, the results of energy‐dispersive X‐ray (EDX) spectrum and mapping (Figure 1d,e) demonstrate the homogeneous presence of MXene sheets on the wool from the distribution of elemental composition (Ti, F, and Cl).

Despite a few aggregates on the surface, a continuous MXene/CNF coating of only 0.92 wt.% (9.7 mg m^−1^) of the yarn exhibited excellent photothermal conversion ability. This surprising performance can be attributed to the remarkable electromagnetic wave absorption ability and the localized surface plasmon resonance effect of MXene.^[^ ^17b^ ^]^ To further evaluate the high efficiency of Ti_3_C_2_T_x_ MXene, the temperature variation and water‐evaporation caused weight loss of the WYM coated with different photothermal agents were recorded with and without NIR light irradiation (Figure S4, Supporting Information). When NIR light was turned on and off, the temperature of the MCWYM increased and declined more obviously than the neat WYM and other WYMs coated with the composite of CNF and carbon black (CB), carbon nanotube (CNT), or reduced graphene oxide (rGO). The rapid temperature change in the MCWYM was attributed to the continuous coating formed by ultrathin MXene nanosheets on the surface of WYMs. The continuous coating can convert NIR light to heat and induce rapid temperature changes of the whole WYM. The trend of weight loss of WYMs coated with different photothermal agents roughly follows their temperature variation profile, confirming the critical role of photothermal effect on water loss (Figure S4c, Supporting Information). The addition of CNF can further improve the photothermal performance of WYM than that coated with neat MXene. The hydrophilic surface and expanded galleries between MXene sheets in the MXene/CNF coating can provide numerous hydrophilic sites for ultrafast water diffusion and reversible water desorption/absorption when light irradiation is switched on/off.^[^ ^21^, ^25^ ^]^ Consequently, the coating of MXene/CNF composite endows the WYM with excellent photothermal effects and fast moisture desorption, favorable for generating a high actuation stroke/stress. Additionally, wool fabric coated with MXene/CNF exhibits excellent photothermal properties (Figure S5a–c, Supporting Information), indicating the potential of MXene/CNF for the large‐scale functionalization of wool textiles. Notably, the moisture permeability of the wool fabric is increased by over 30% after coating with MXene/CNF (Figure S5d, Supporting Information) due to the high hygroscopicity of the coating, highlighting the significant advantage of MXene/CNF functionalization in enhancing the comfort of wool textiles.

### 2.2 Self‐Plied MCWYM with High Contractile Actuation Stress

**Figure 2:**
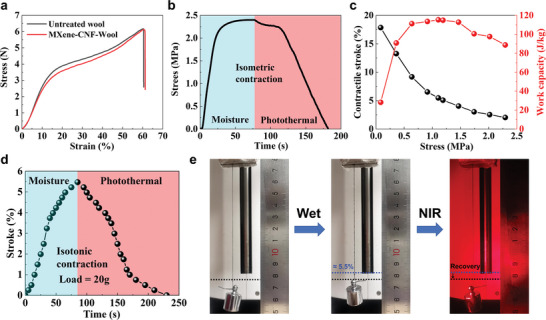
Mechanical and actuation performances of self‐plied MCWYMs. a) Tensile stress–strain curves of the neat WYM and MCWYM. b) Isometric actuation stress of the self‐plied MCWYM under moisture exposure and NIR light irradiation. c) Dependence of contractile stroke and work capacity of the self‐plied MCWYM on the applied stress upon moisture exposure. d) Isotonic contractile stroke and e) photos of the self‐plied MCWYM under a load of 1.11 MPa (20 g) under moisture exposure or NIR light irradiation.

The mechanical properties of yarn muscles are the basis of their actuation performance in artificial muscle applications. As shown in **Figure** **2**a, the MXene/CNF coating does not impair the tensile properties of the self‐plied WYM, with a maximum strength of more than 6.1 MPa for the coated yarn muscle, which shows much higher strength than the human skeletal muscle (0.3 MPa).^[^ ^26^ ^]^ The isometric contractile force/stress of the resulting MCWYM was monitored by a static mechanical tester at a fixed gauge length while it was exposed to the water fog or NIR light (700 mW cm^−2^). For ease of expression, the stress was calculated as the weight of the load divided by the cross‐sectional area of the self‐plied MCWYM.^[^ ^6a^ ^]^ For a better understanding, the mass of the load was also used directly in place of the stress (Figure S6, Supporting Information). As shown in Figure 2b, maximum contractile stress with 2.4 MPa was achieved when it was exposed to moisture and remained there as long as the MCWYM was kept wet. When water fog was stopped and NIR light irradiation was commenced, the contraction force started to decrease slowly first and then sharply and finally returned to zero, which indicates the yarn muscle underwent relaxation and completed the fully reversible cycle. Notably, the maximum contraction stress of the MCWYM was much lower than its yield point (≈3.15 N, shown in Figure 2a), which means the maximum contraction stress does not exceed the elastic limit so that the MCWYM can undergo reversible actuation.

Based on the results of the isometric contraction, we tested the isotonic contraction stroke of a self‐plied MCWYM of ≈10 cm long under different loads below 2.4 MPa during its exposure to water fog. The weightlifting performance under these loads was quantified by its contractile stroke and work capacity. As shown in Figure 2c, the contractile stroke decreased almost monotonically with the increase of applied stress. The yarn muscle performed a large contractile stroke of 17.86% when the load was 0.8 MPa (1.5 g). It is worth mentioning that the yarn muscle can lift a load of 2.29 MPa (41.5 g, more than 3400 times its own weight) by ∼2.03%. The work capacity increased to the maximum value of 115.34 J/kg at an applied stress of 1.11 MPa (20 g) and then decreased with further increase of the applied stress. It was found that under this 20‐g load, the WCWYM can achieve 5.47% contraction stroke under moisture exposure and complete recovery quickly under NIR light (Figure 2d,e).

This contractile actuation of MCWYM can be attributed to the hygroscopic volume expansion of wool fibers and the twisted self‐plied structure of WYM (Figure 1a; Figure S1, Supporting Information). A single wool fiber can produce more than 16% radial expansion after absorption of moisture, corresponding to about 40% volume expansion.^[^ ^15a^ ^]^ When the wool fibers became wetted, their volume expansion caused a radial swelling of the self‐plied WYM, which also represents the increase in the spiral path of fibers. In the self‐plied structure with stable mechanical self‐locking character (Figure S1, Supporting Information), the wet fibers that were not axially elongated had to match the larger spiral path, which eventually caused the shrinkage of the yarn in the length direction and provided the contractile force. Concomitantly, the individual fiber segments would untwist by hygroscopic swelling, which would insert twists into the self‐plied structure due to the opposite direction of individual fibers and the self‐plied wool yarn. So, the yarn muscle would also rotate during the contraction (Movie S1, Supporting Information), and physically, this rotation could also contribute to the contractile actuation.^[^ ^27^ ^]^ And under NIR light illumination, the rapid water loss in the MCWYM resulted in the relaxation of contraction and the release of actuation, which was driven by the opposite morphological variation of fibers.

### 2.3 Photothermal Actuation of Coiled MCWYMs with Large Stroke

**Figure 3:**
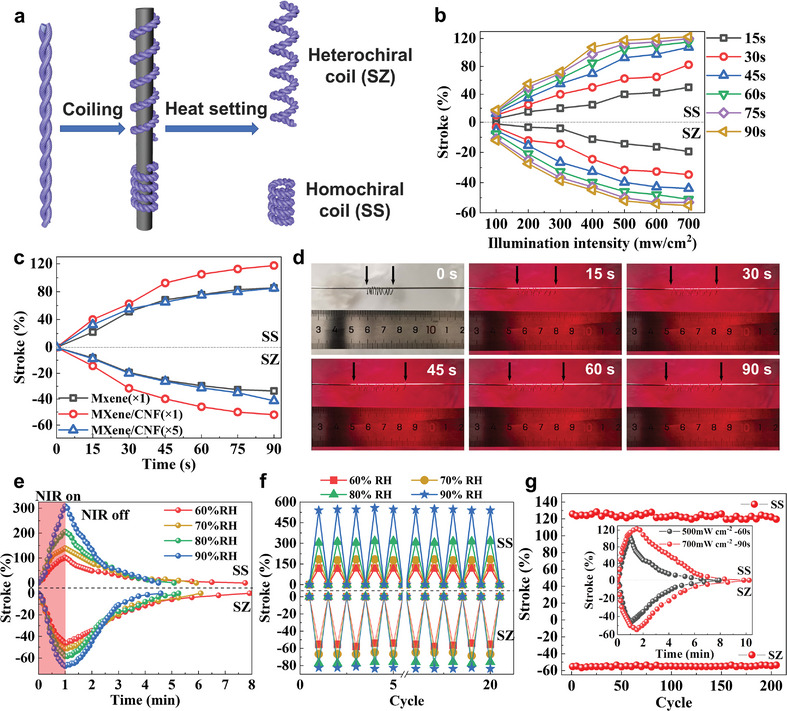
Fabrication and photothermal actuation of coiled MCWYMs. a) Schematic illustration of the fabrication of MCWYMs with homochiral and heterochiral coils. b) Stroke of coiled MCWYM under different NIR light intensity for 15–90 s. c) Stroke of coiled WYM with different MXene‐based coatings under 500 mW cm^−2^ NIR light. d) Photographs of the homochirally coiled MCWYM before and after NIR irradiation (500 mW cm^−2^) for different durations. e) Photothermal actuation stroke under 500 mW cm^−2^ NIR irradiation and recovery of coiled MCWYMs under different RH. f) Change in the actuation stroke of coiled MCWYMs during the initial 20 cycles with 700 mW cm^−2^ NIR irradiation (90 s irradiation for each cycle) at different RH. g) Change in the maximum actuation stroke (cycling stability) of the coiled MCWYMs at different actuation cycles under 700 mW cm^−2^ NIR irradiation (90 s irradiation for each cycle) and 60% RH.

In addition to the high‐stress actuation, the large‐stroke actuation under wireless NIR light stimuli is also important for the application of the MCWYMs. We further processed the self‐plied MCWYMs into spring‐like structures via coiling (**Figure** **3**a; Figure S7, Supporting Information). As shown in Figure 3a, the self‐plied MCWYM was wrapped over a mandrel to form a chiral coil, and subjected to annealing at 120 °C for 10 min to obtain a coiled MCWYM with large‐stroke photothermal actuation. The matching or opposition of the chirality between the individual fiber and the coil will determine the actuation direction of these artificial muscles under NIR light. When the chirality of the individual fiber is same (homochiral: SS) as or opposite (heterochiral: SZ) to the coil's chirality, the coils will undergo photothermal expansion or contraction respectively.

Coiled MCWYMs unlike the above uncoiled MCWYM with one or two fixed ends, the coiled MCWYMs have another operation mode with both ends allowed to move axially under NIR light irradiation, exhibiting a large expansion or contraction stroke. For convenience, unless otherwise specified, the coiled MCWYMs were characterized without torsional tethering and any load under 20 °C, 60% relative humidity (RH). The stroke of coiled MCWYMs under different NIR light intensity for 15–90 s are shown in Figure 3b. It is found that for both chiral coiled MCWYMs, the stroke markedly increased with rising NIR illumination intensity from 100 to 500 mW cm^−2^, and then gradually became mild, which means that most of the adsorbed water in the yarn muscle was quickly removed when the illumination intensity exceeded 500 mW cm^−2^. Then the photothermal actuation strokes of coiled WYMs with different coating processes, that is, coated with MXene (one time), MXene/CNF(one time), or MXene/CNF (five times) were compared under different light intensity (100, 500, and 700 mW cm^−2^) for 15–90 s. As shown in Figure 3c and Figure S8 (Supporting Information), the one‐time MXene/CNF‐coated WYMs performed higher actuation strokes than the MXene‐coated counterpart under the intensity of either 500 or 700 mW cm^−2^. This is attributed to that the added CNF could create many nanochannels between the MXene nanosheets and result in more moisture absorption in the coating and faster water desorption from the yarn under NIR light irradiation, leading to improved actuation speed and stroke of the yarn muscle. The 5‐times‐coated WYMs showed the best actuation performance than other coated WYMs under the NIR light irradiation of 700 mW cm^−2^, but the worst under 100 mW cm^−2^. It is probably due to that the multiple MXene/CNF coating could allow the coated WYM to hold more moisture at ambient state but also require more energy to remove this moisture for improving the stroke. Therefore, under comprehensive consideration, 1‐time MXene/CNF coating was the best choice as it can balance the stroke and response time. Figure 3d shows the image records of a homochirally coiled MCWYM under 500 mW cm^−2^ NIR light for every 15 s. The coil elongated quickly during the first 45 s and achieved a stroke of 117.5% after 90 s, which was induced by the distinct water desorption of WYM caused by the excellent photothermal conversion ability of MXene. Further evidence of the significant contribution of the MXene/CNF coating to the actuation of WYM is shown in Figure S9 (Supporting Information). After 60 s NIR irradiation, the homochirally and heterochirally coiled pure WYM (without MXene/CNF coating) only achieved 16.67% elongation and 12.62% contraction, respectively, which are much lower than that of homochirally and heterochirally coiled MCWYMs (103.03% elongation and 46.15% contraction, respectively) under the same conditions. Despite more water loss under NIR irradiation, the MCWYMs recovered as fast as the pure WYMs when the NIR light was off. These results indicate that the MXene/CNF coating serves as both the photothermal and hygroscopic agent, accelerating water evaporation under NIR light and moisture absorption in ambient air. It is worth mentioning that our device can also produce similar photothermal actuation under natural sunlight. As shown in Figure S10 (Supporting Information), the MCWYM homochiral coil achieved an elongation of 76.47% after being exposed to sunlight for 90 s, which proves the practicality of our device in the natural environment.

Considering the different atmospheric humidity and the important role of moisture in the photothermal actuation of MCWYMs, it is necessary to investigate their performance of coiled MCWYMs under different RH. It was found that the actuation stroke of heterochirally and homochirally coiled MCWYMs both increased monotonically with the RH increase (Figure 3e). When the RH increased to 90%, an expansion of more than 300% and a contraction of almost 70% was respectively obtained for the heterochirally and homochirally coiled MCWYMs under 500 mW cm^−2^ NIR light for 60 s. The actuation of coiled MCWYMs was fully reversible as shown by the gradual recovery after NIR light was off. The higher humidity environment allowed the yarn muscles to absorb water faster, so it took less time to recover. In the higher humidity environment, the yarn muscle can absorb more water to reach the equilibrium state, after which it would take a longer time to absorb more thermal energy to remove the water. In addition, the stroke of coiled MCWYMs could be further enhanced by increasing the NIR illumination intensity and duration. Figure 3f illustrates the reversibility of coiled MCWYMs during the initial 20 cycles of actuation under 700 mW cm^−2^ NIR irradiation for 90 s, including the strokes at the initial state, maximum actuation, and recovered state for each cycle. The stroke of all samples was further improved under increased NIR irradiation, and a larger contraction (over 83%) and elongation (about 550%) achieved for the heterochirally and homochirally coiled MCWYMs under 90% RH. The comparison of the contraction stroke (most previous research on yarn muscles focused on contractile actuation) and the response rate with other reported humidity‐ or photothermal‐responsive artificial muscles is presented in Figure S11 (Supporting Information). The maximum moisture‐induced stroke (regarded as equal to the photothermal elongation stroke) of our coiled MCWYMs is much higher than that of other naturally derived fiber‐based muscles, and the photothermal contraction stroke was also comparable to other artificial muscles. Additionally, the moisture actuation rate of our coiled MCWYMs (0.94% s^−1^) is currently the highest among natural‐fiber‐based artificial muscles, and the photothermal actuation rate (0.92% s^−1^) is also superior to most similar fiber‐based artificial muscles. Both homochirally and heterochirally coiled MCWYMs show excellent reversibility of photothermal actuation, and the recovery behavior after irradiation with 700 mW cm^−2^ – 90 s NIR light was similar to that after with 500 mW cm^−2^ −60 s condition (Figure 3g inset). Such yarn muscles also show a long actuation life with more than 200 cycles (Figure 3g), which has far exceeded the cycles of similar natural fiber‐based long‐stroke coils (with only 10–50 cycles).^[^ ^19^, ^27^, ^28^ ^]^ Furthermore, the photothermal actuation force generated by the elongation of the homochirally coiled MCWYM (SS) was further evaluated. The homochiral WYM coil can attain a blocking force that is equivalent to the pressure over 1 KPa during the NIR light irradiation of 700 W cm^−2^ for 40 s (Figure S12, Supporting Information). The excellent actuation performances of our coiled MCWYMs demonstrate the advantages clearly compared to other reported artificial muscles with similar working modes.

### 2.4 Actuation Mechanism of Coiled MCWYMs

**Figure 4:**
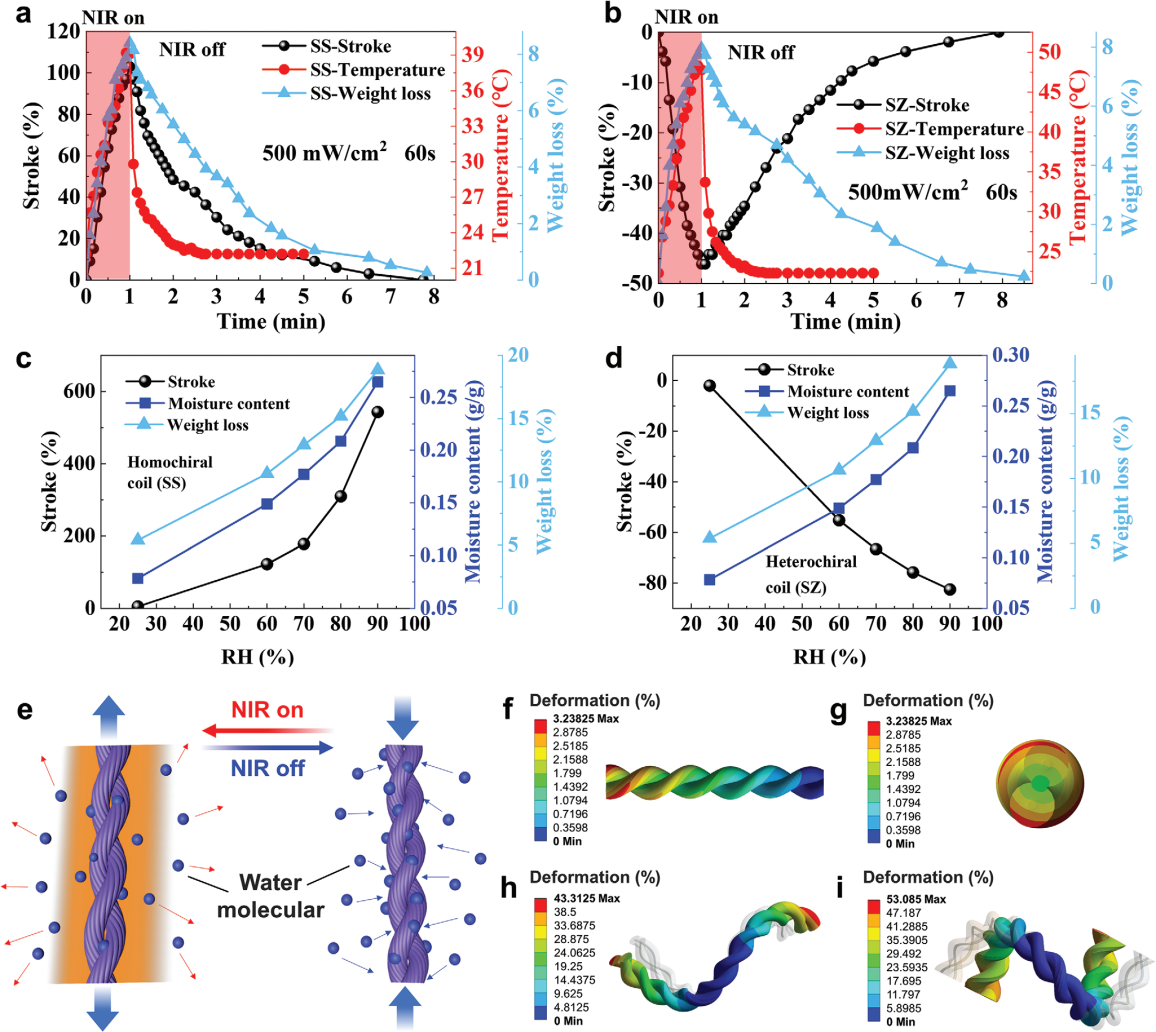
Mechanism studies of the coiled MCWYMs. a,b) Changes of weight loss, temperature, and stroke during the actuation and recovering process of the MCWYMs with homochiral (a) and heterochiral coils (b). c,d) Dependence of the stroke, moisture content, and weight loss of MCWYMs with (c) homochiral and (d) heterochiral coils on the ambient RH. e) Illustration of the actuation mechanism of the MCWYMs. f–i) Finite element simulation results of the photothermal actuation of self‐plied MCWYMs with frontal (f) and cross‐sectional view (g), and the homochirally (h) and heterochirally (i) coiled MCWYMs. The grey profiles in h and i indicate the initial states.

**Figure** **4**a,b depicts the weight loss, temperature change, and the dynamic actuation stroke of coiled MCWYMs exposed to 500 mW cm^−2^ NIR for 60 s and the recovery under 60% RH. The coiled MCWYMs could recover 90% of the photothermal stroke after 4 minutes under 60% RH. For the actuation process, the weight loss, temperature change, and stroke all increased rapidly over time in the similar trend, and during the recovery process, the temperature decreased more quickly after NIR was switched off, and the trend of weight loss was basically the same as the stroke was changed. This observation indicates that water loss is a more influential factor for actuation compared to temperature changes. This synchronicity of water loss with actuation of homochirally/heterochirally coiled MCWYMs also demonstrates why the photothermal actuation of the yarn muscles was fully reversible. When the coiled MCWYMs were exposed to water fog immediately after the NIR light was off, they recovered to their initial states within 20 s (Figure S13, Supporting Information), because they can quickly absorb a large amount of moisture provided by water fog and initiate a moisture‐driven actuation that was opposite to the previous photothermal actuation. This result further supports that the release and absorption of moisture play a pivotal role in the actuation of coiled MCWYMs. Consequently, the reversible actuation mechanism of coiled MCWYMs should be considered relying on the moisture loss and gain caused by the photothermal conversion effect and the hygroscopicity of wool fibers and MXene/CNF coating, respectively.

We established the relationship of water content in the initial state, the weight loss after 700 mW cm^−2^ NIR illumination for 90 s, and the corresponding stroke of the homochirally (Figure 4c) and heterochirally (Figure 4d) coiled MCWYMs at different RH. The results show that in the higher RH environment, the coiled MCWYMs contained more moisture in the initial state and achieved a larger stroke, and the water loss during NIR illumination was also more. Thereby, the NIR illumination and moisture were considered as the two fundamental factors governing dual‐responsive MCWYM. The trend of weight loss of these yarn muscles was basically the same as the stroke change under different RH, indicating that the release and absorption of water was the key cause for the photothermal actuation of the coiled MCWYMs. The trends of their stroke and temperature rise against the NIR light power density were also recorded. As shown in Figure S14 (Supporting Information), the stroke and temperature rise markedly increased with rising NIR light power density from 100 to 900 mW cm^−2^, and the temperature data set can be fitted into polynomial extrapolation with a R^2^ >0.995. The MCWYMs performed steadily increased actuation in relatively low temperature states when the power density was increased from 100 to 500 mW cm^−2^, after which the temperature rise accelerated as the power increased, but the curve of stroke became flattened. It indicates that the change of water content was more suitable than that of temperature for evaluating the overall actuation performance for coiled MCWYMs. We further used X‐ray diffraction (XRD) to monitor the microstructural evolution of MCWYM under moisture exposure or NIR illumination. Following NIR irradiation, the characteristic peaks of keratin at 9.4° and 20.6° (both ascribed to the α‐helix chain and β‐keratin crystalline in the protofibril, respectively)^[^ ^29^ ^]^ shift to 9.6° and 19.1°, and become stronger due to water evaporation. Upon moisture absorption, these peaks relocate to 9.2° and 27.5° and become much weaker (Figure S15, Supporting Information). The insertion/removal of water molecules in the amorphous matrix hosting the protofibril leads to the increase/decrease in the background, thus changing the total intensity of these two peaks. The shift of these two peaks under the NIR or moisture environment can be attributed to the insertion/removal of a small amount of water molecules in the protofibril. These findings solidly confirm the substantial and reversible nature of moisture insertion/removal at the hydrophilic sites, triggering the volume changes in the fiber that are reflected macroscopically as the dual‐responsive actuation of MCWYM. As illustrated in Figure 4e, under NIR light, upon desorption of water molecules, each individual fiber with a S twist would continue to increase rotation because of the volume shrinkage, which would produce the torque and lead to the decrease of the plying of the two yarns in the Z direction due to the direction of plying was opposite to the fiber twist direction.^[^ ^30^ ^]^ At the same time, this rotation will also drive the coil structure to elongate or contract according to the homochirality or heterochirality of the coil.^[^ ^31^ ^]^ After the NIR light is switched off, the water adsorption of coiled MCWYMs causes these processes to reverse.

To further understand the actuation process, the deformation distribution of the coiled MCWYMs actuator was investigated and predicted by the finite element analysis (FEA). Considering that wool yarn is a complex system with intricate composition and multi‐scale structure, the FEA method stands out as one of the most efficient tools for analyzing and simulating the mechanics of these fiber assemblies.^[^ ^32^ ^]^ A double‐wound spring model with the characterized parameters being assigned has been developed, and the symmetrical constraints for the boundary conditions and the displacement were also implemented. The simulation for the self‐plied MCWYM (Figure 4f–g) reveals that the macroscopic photothermal actuation along the axial direction was a result of the untwisting rotation induced by the volume changes. Based on this insight, the simulation for the coiled MCWYM was further explored. It was found that the deformation and rotation of the self‐plied structure further caused the rotation of the coil structure, resulting in a macroscopic expansion/contraction for the homochiral/heterochiral coil. As shown by the modeling results (Figure 4f–i; Movie S2, Supporting Information), its 3D architecture (color scheme) matches with the experimental result very well, the coiled MCWYMs can also reversibly perform elongation or contraction. Under NIR light, each fiber segment shrunk in volume as desorption of water molecules, which produced the torque for self‐ply yarn and caused further deformation of the coils. From the modeling, the reversible elongation or contraction of coiled MCWYMs could be mainly attributed to the volume changes of the fibers and plying yarns caused by NIR irradiation, which means the actuation could be controlled by adjusting the relative volume expansion or contraction.

### 2.5 Applications of the MCWYMs

**Figure 5:**
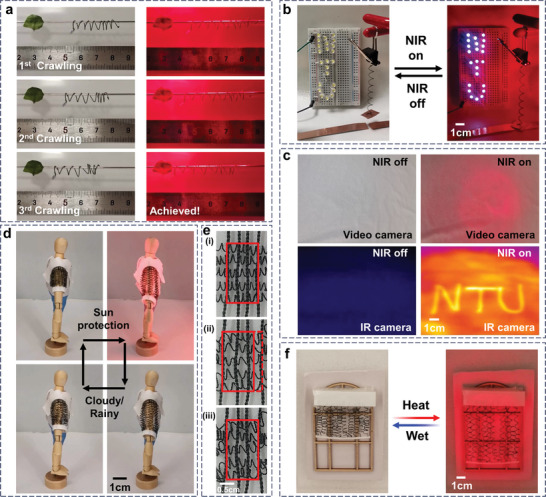
Demonstrations of potential applications based on MCWYMs. a) Sequential photos showing the caterpillar‐like robot crawling progressively, which was alternatively driven by moisture (left column) and NIR light irradiation (right column). b) Photographs of a smart switch based on the MCWYM with homochiral coils. c) Photographs and IR images of a patterned textile for information encryption and display before and after NIR irradiation. d) Sequential photos of a smart sleeve based on homochirally coiled MCWYMs, which responds to the weather change. e) Photographs showing the pore changes in a smart textile woven from heterochirally coiled MCWYMs, under i) initial state, ii) exposure to moisture, and iii) recovered state. f) Photographs of the smart curtain based on homochirally coiled MCWYMs, spreading out on sunny days and rolling up on cloudy/rainy days.

The wireless NIR responsiveness property of the MCWYMs can further demonstrate the potential eventual use‐cases. Various smart devices were being developed by integrating these MCWYMs actuators, such as smart switch, crawling caterpillar‐like robot, smart sleeves, smart window shade, and information encryption textile (**Figure** **5**). In nature, the crawling of the caterpillar is not only derived from the peristalsis of its legs, but more importantly, the tension‐based mechanism,^[^ ^33^ ^]^ that is, the caterpillar changes its shape and induces movement through the asymmetric expansion and contraction of different parts of its body.^[^ ^34^ ^]^ Inspired by this crawling mechanism of the caterpillar, we prepared a robotic caterpillar made from yarn muscle. Under the sequential biased stimulation of NIR light and moisture fog, the robotic caterpillar expanded and contracted recurrently to approach a leaf gradually. NIR irradiation was biased toward the end of the caterpillar near the leaf, while moisture fog was biased toward the other end. Consequently, whenever the robotic caterpillar was stimulated by NIR light or moisture, it crawled toward the leaf. This robotic caterpillar can move more than 7 mm with one walking cycle and achieve the goal after three crawls (average speed: 3.5 mm min^−1^), as shown in Figure 5a and Movie S3 (Supporting Information).

It is noteworthy that compared with crawling around a metal rod, crawling on a rough surface is more challenging because of the larger contact area. Our robotic caterpillar is capable of crawling effectively even on a rough surface. Figure S16a and Movie S4 (Supporting Information) show that the robotic caterpillar successfully crawled over 6 mm on a rough sandpaper surface during a single crawl (average speed: 2.32 mm min^−1^). In addition, crawling on a smooth surface is typically considered extremely difficult for crawlers, but our robotic caterpillar accomplished this task with ease. As illustrated in Figure S16b and Movie S5 (Supporting Information), the robotic caterpillar directionally moved more than 5 mm on the smooth glass after two crawls, (average speed: 1.31 mm min^−1^). These results demonstrate that the crawling of our MCWYM‐based robotic caterpillar is not highly dependent on friction, in contrast to most two‐anchor crawlers. The asymmetric tension‐based mechanism, similar to the natural caterpillar, is introduced by the different biases of the NIR light and moisture stimuli, enabling the directional crawl of our robotic caterpillar on a smooth surface. The ability to crawl on both smooth and rough surfaces highlights the adaptability of our actuator to different substrates, opening up a range of potential applications.

We also demonstrated a wireless NIR light controlled smart switch by the MCWYMs actuator (Figure 5b; Movie S6, Supporting Information). A MCWYM homochiral coil was constructed as a photothermal electrical switch, which connected the electric circuit under NIR illumination and disconnected the circuit after NIR light was turned off. The photothermal actuation behavior of the coiled MCWYM switch was also demonstrated on a circuit comprising the light‐controlled switch in series with the light‐emitting diode (LED). The LED could not be lit without NIR light irradiation. Meanwhile, the coiled MCWYM elongated and directed the conductor processing circuit under NIR light irradiation, resulting in the LED to be successfully lighted up at the same time. When the NIR light turned off, the contraction of the coiled MCWYM caused the circuit to open and the LED to be switched off.

Considering the excellent photothermal performance of the MXene/CNF, we can convey the information by local photo‐heating of the pattern through NIR light. Here, we illustrate patterns with MCWYM and use it as the inner lining of ordinary fabrics. This preparation procedure can fabricate any desired pattern that is scalable and at a low cost. As shown in Figure 5c and Movie S7 (Supporting Information), due to the apparent temperature difference caused by the excellent photothermal properties of MXene, the word “NTU” can be IR camera readable with the NIR light locally irradiating the pattern. However, it was invisible to the human naked eye, with or without NIR illumination. In this way, the information carried by the WYM could be encrypted and displayed successfully.

Weather changes will directly affect the thermal and humid comfort of the human body, and wool textile provides outstanding style and comfort. Here, we presented a smart textile made by MCWYMs, which responded to weather changes in the environment as shown on the wooden mannequin. These sleeves extend to play a role of sun protection under the NIR light irradiation (sunny sunshine mode), and with the NIR light off and encountering moisture, the sleeves automatically roll up and recover to half sleeves (cloudy or rainy‐day mode). This deformable light‐responsive textile holds promise for efficient adaptive moisture and heat management of human comfort (Figure 5d; Movie S8, Supporting Information). Apart from external weather changes, variations in human physiological states (such as exercise, sleep, excitement, etc.) necessitate diverse different heat/moisture comfort requirements. To address this challenge, we developed a smart textile using heterochirally coiled MCWYMs, which adaptively regulate pore size to accommodate individualized heat/moisture comfort management. As shown in Figure 5e and Movie S9 (Supporting Information), the pores of the textile expand upon moisture absorption. Specifically, after being wet for 90 seconds, the pore area increased by more than 27% (Figure 5e‐i,ii). This mimics the scenario when the human body sweats, the smart textiles could enhance convection, radiation, and evaporation by enlarging the pores, thus facilitating the dissipation of body heat. Subsequently, as moisture‐wetting ceases, the pores gradually revert to their original size. This self‐adaptive smart textile effectively achieves heat/moisture comfort management by dynamically changing its microstructure, enhancing comfort between human skin and fabric.

Other applications of light‐responsive WYM can be in home textiles or engineering fields. For example, greenhouses generally need to use sunshade fabrics to tune the illumination and temperature inside. A smart window shade had been developed by using the MCWYM homochiral coils (Figure 5f; Movie S10, Supporting Information). This smart window shade can spread out on the window on sunny days to prevent the scorching sunshine from entering the greenhouse. In the case of cloudy and rainy days with weak light, the smart shade can be rolled up to let in more sunlight. Then, such smart textiles based on WYM can be applied not only to wearable devices, but also to industrial textiles and even smart agriculture applications.

## 3 Conclusion

In conclusion, a dual‐responsive artificial muscle was developed and established using MXene and wool yarn, and its applications were extended to several fields requiring high output force and large deformation. The MCWYMs were successfully fabricated by a convenient approach, and the WYM with both two operation modes featured strong figures of merit and capabilities under moisture and wireless NIR light stimuli. The actuation mechanisms are also investigated in detail by using the finite element simulation. The contractile WYM could lift more than 3400 times its own weight, and the NIR light responsive coiled MCWYMs could provide more than 83% contraction or 550% elongation. The actuation of WYM was considered to be a result of water molecules deintercalation/intercalation induced by the photothermal conversion of MXene and volume shrinkage/expansion of wool fibers. These actuators could also be designed for adaptive integration and programmable actuation, and provide opportunities for potentially promising applications, such as smart electrical switch and soft robotics. Moreover, these explored devices also present novel possibilities for the self‐adaptive smart textile, intelligently adjusted greenhouse, and the information encryption/display camouflage textiles. Ultimately, the explorations of these MCWYMs open a new horizon for a number of tunable functionalities and are desirable to benefit numerous aspects in our daily life and engineering fields.

## Conflict of Interest

The authors declare no conflict of interest.

## Author Contributions

L.Z. and S.C. contributed equally to this work. P.S.L. and L.Z. conceived and designed the project. L.Z. conceived the devices and, carried out most of the experiments, characterizations, and data analyses. S.C. gave advice on the experimental design and mechanism and data analyses and performed the synthesis of MXene and EDX characterization. Y.X. carried out the theoretical simulations and calculations. N.W. contributed to the selection of wool. J.L., H.F., D.G., F.J., and X.Z. gave suggestions on the experiments and data presentation. L.Z. and S.C. wrote the manuscript. P.S.L. and N.W. supervised this project. All authors provided comments and agreed with the final manuscript.

## Supporting information

Supporting Information

Supplemental Movie 1

Supplemental Movie 2

Supplemental Movie 3

Supplemental Movie 4

Supplemental Movie 5

Supplemental Movie 6

Supplemental Movie 7

Supplemental Movie 8

Supplemental Movie 9

Supplemental Movie 10

## Data Availability

The data that support the findings of this study are available from the corresponding author upon reasonable request.
