# Supplementary material for: Dual‐Responsive MXene‐Functionalized Wool Yarn Artificial Muscles
Source: Adv Sci (Weinh). 2024 Apr 22;11(25):2402196. doi: 10.1002/advs.202402196 (PMC11220689; doi:10.1002/advs.202402196)
Supplement: Supplementary file 1 — Supporting Information [file ADVS-11-2402196-s008.pdf]

ADVANCED SCIENCE
Open Access

Supporting Information

for *Adv. Sci.*, DOI 10.1002/advs.202402196

Dual-Responsive MXene-Functionalized Wool Yarn Artificial Muscles

Liuxiang Zhan, Shaohua Chen, Yangyang Xin, Jian Lv, Hongbo Fu, Dace Gao, Feng Jiang, Xinran Zhou, Ni Wang and Pooi See Lee**

Supporting Information

Dual-responsive MXene-functionalized wool yarn artificial muscles

Liuxiang Zhan, Shaohua Chen, Yangyang Xin, Jian Lv, Hongbo Fu, Dace Gao, Feng Jiang, Xinran Zhou, Ni Wang* and Pooi See Lee*

Contents

Supporting Text
Figures S1 to S16

Other supplementary materials for this manuscript include the following:

Movies S1. Video of a self-plied MCWYM under a load of 1.11 MPa (20 g) under moisture exposure and then NIR irradiation.
Movies S2. Video of simulated photothermal actuation of coiled MCWYMs by finite element analysis.
Movies S3. Video of a caterpillar-like robot crawling progressively, which was alternatively driven by moisture and NIR light irradiation.
Movies S4. Video of a caterpillar-like robot crawling progressively on a rough sandpaper, which was alternatively driven by moisture and NIR light irradiation.
Movies S5. Video of a caterpillar-like robot crawling progressively on a smooth glass, which was alternatively driven by moisture and NIR light irradiation.
Movies S6. Video of a smart switch based on the homochirally coiled MCWYM.
Movies S7. Video of a patterned textile with self-plied MCWYM for information encryption and display before and after NIR irradiation.
Movies S8. Video of a smart sleeve based on coiled MCWYMs responding to the weather change.
Movies S9. Video of a pore changing smart textile woven from MCWYMs responding to the moisture.
Movies S10. Video of a smart curtain based on coiled MCWYMs responding to sunny days and cloudy/rainy days

Materials and Methods

Preparation of MXene/CNF and nanocarbon/CNF dispersions

$Ti_3C_2T_x$ MXene nanosheets were synthesized from Ti_3C_2Al powder by etching Al according to a modified "minimally intensive layer delamination" method, as our previous studies mentioned.[25] 1.5 g LiF and 1 g Ti_3C_2Al were dispersed in 5 mL of deionized water by stirring, then 15 mL of 12 M HCl was added into the suspension gradually under continuous stirring. The mixture was allowed to react at 35 °C for 36 h under magnetic stirring. After that, the reaction mixture was centrifuged to give the etched precipitate, which was repeatedly washed with deionized water and centrifuged for 5 times until the pH of the supernatant was around 5. The final precipitate was added to 300 ml deionized water and purged with Ar for 10 minutes, and then ultrasonicated in an ice/water bath for 1 hour. The resulting suspension was centrifuged at 3000 rpm for 30 min, and 80% of the supernatant was collected as a dispersion of $Ti_3C_2T_x$ MXene nanosheets. The MXene dispersion was mixed with a CNF aqueous dispersion at a weight ratio of 10:1 under ultrasonication for 10 minutes, and the resulting mixture was concentrated by rotary evaporation at 20°C to give a MXene/CNF dispersion of 0.5 wt %.

Fabrication of MXene/CNF- functionalized WYMs and fabrics

The wool yarns (Jiangsu Danmao Textile Co., Ltd. China) with a diameter of ~ 240 μm were all washed by a nonionic detergent solution to enhance the hydrophilicity before using. A neat WYM was fabricated as illustrated in Figure 1a. Typically, a wool yarn was suspended between an electric motor and a load that can move vertically but not rotationally, then twists were inserted to the yarn by the rotating motor (twist density of the wool yarn was 2000 turns m^{-1}). During this process, the wool yarn continuously shortened and lifted the load, then the load was moved to the midpoint of the twisted yarn which was following folded, the single yarn untwisted from the individual fiber and created a twist for the double yarn, and finally forming a stable self-plied WYM. Such self-plied WYM was immersed in the aforementioned MXene/CNF mixture dispersion for 10 min and supplemented with ultrasonication, then it was hung in the air for drying at room temperature for 12 h to obtain a self-plied MXene/CNF-coated WYM (MCWYM) with high actuation stress. Following this coating procedure, the loading of MXene/CNF composites was 0.92 wt% (9.7 mg m^{-1}) in the self-plied MCWYM. To fabricate a large-stroke coiled muscle (see Figure 3a), a self-plied MCWYM, that has been coated with MXene/CNF, was wound on a stainless-steel mandrel with its two ends being fixed (the spring index for MCWYM coil was 8.4), then it was thermally set at 120 °C for 10 min under vacuum. Since the actuation characteristics of the coil yarn muscles depended on their chirality, different spiral directions were selected when the MXene/CNF-coated self-plied WYM were wound on the mandrel to finally obtain the homochiral/heterochiral coiled WYM. The wool fabric coating process mirrors that of MCWYM. The wool fabric was soaked in the MXene/CNF mixture dispersion for 10 minutes, assisted by ultrasonic waves. Subsequently, it was hung in the air at room temperature for 12 hours, resulting in a wool fabric with MXene/CNF functionalization (loading of MXene/CNF composites was 1.15 wt%).

Characterizations

The morphology of the WYMs and MCWYMs was characterized by a field-emission scanning electron microscope (FESEM, JEOL 6340F). The element analysis was performed on a FESEM (JEOL 7600F) with an EDX analyzer. The mechanical properties were measured using a Materials Testing System (Model 43, MTS Systems Corporation) with a 500-N load cell at ambient temperature. The blocking force of a homochiral MCWYM coil under NIR irradiation was monitored by placing its tip against a digital balance (ME204, Mettler Toledo). Light absorbance was measured on a spectrophotometer (UV-1600PC, MAPADA). Attenuated total reflectance Fourier-transform infrared spectroscopy (ATR-FTIR) was performed on a PerkinElmer Frontier spectrometer. The water fog generator with a spraying speed of 1.5 g min^{-1} was used for moisture generation. The change in crystalline and amorphous phases of MCWYM was monitored on an X-ray diffractometer (Bruker D8 ADVANCE). The moisture permeability of fabrics was evaluated using a moisture permeability tester (YG601H, NBFY).

Calculation details

The actuation strokes of MCWYMs were calculated by the following formula:

$$\text{actuation stroke (\%)} = \frac{(\mathrm{L} - L_0)}{L_0} \times 100\% \tag{S1}$$

where L_0 is the initial length, and L is the length after actuation.
The work capacity (C) per mass of the WYM was calculated by the equation:

$$C = \frac{Mg\Delta H}{m} \tag{S2}$$

where M is the mass of load, g is the gravitational acceleration constant, ΔH was the change of height, m was the mass of the MCWYM.
The moisture content (MC) was defined as:

$$MC = \frac{M_e - M_0}{M_0} \tag{S3}$$

M_e and M_0 are the wet weight (g) and the dry weight (g) of the MCWYM respectively.

Supplementary Figures

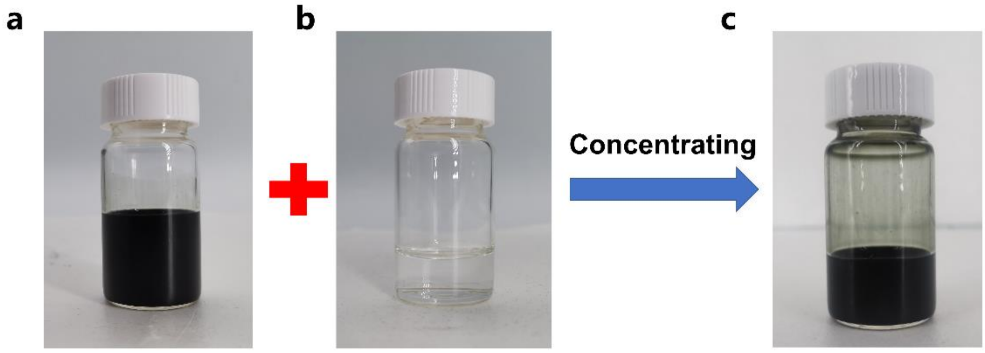

Figure S1. Preparation of a MXene/CNF dispersion. (a, b) Photos of aqueous dispersions of (a) MXene and (b) CNF both with a concentration of 0.5 wt%. (c) Photo of a MXene/CNF composite dispersion (weight ratio of MXene/CNF = 10:1).

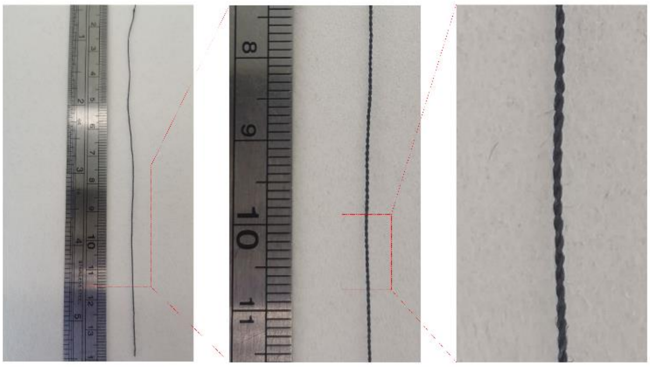

Figure S2. Photographs of a self-plied MCWYM.

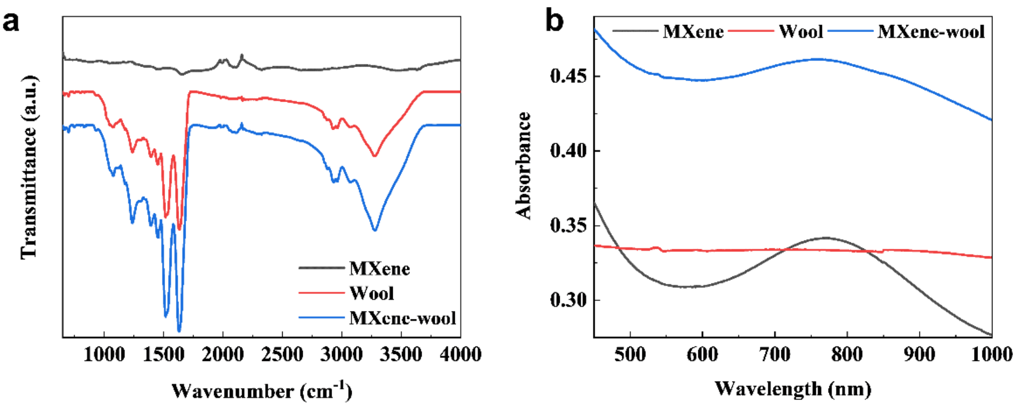

Figure S3. Optical spectra of MXene, wool yarn, and MXene-coated wool yarn. (a) FTIR spectra. (b) UV-Vis spectra. Compared with neat wool yarn, the MXene-coated wool yarn shows an increased light absorbance in both visible and NIR regions with a peak at 780 nm.

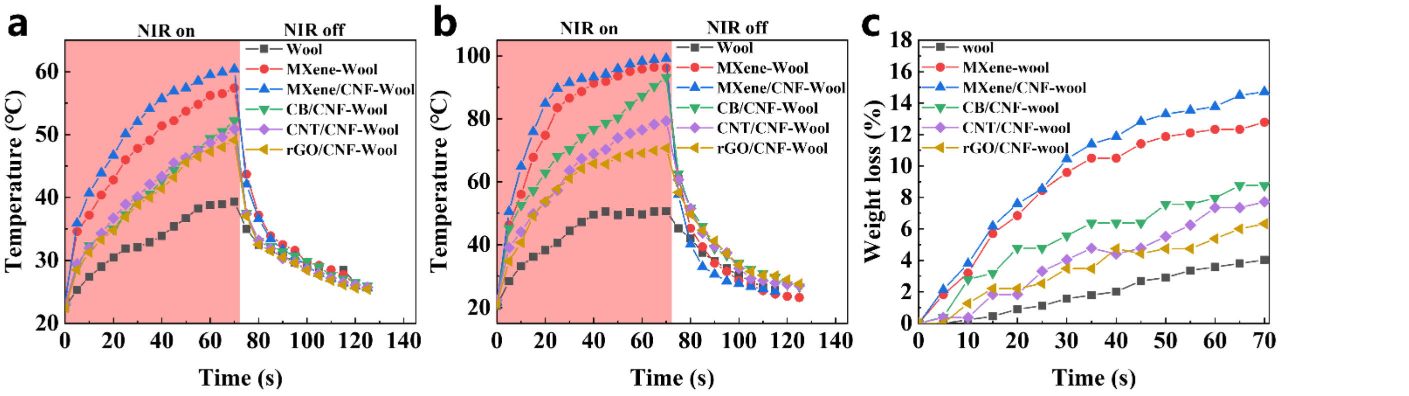

Figure S4. Photothermal performance of the WYMs coated with different photothermal agents. (a, b) Temperature change profile as the (a) 700 mW cm^{-2} and (b) 1.25 W cm^{-2} NIR light was turned on (for 70 s) and off. (c) Weight loss as a function of time under 700 mW cm^{-2} NIR light (70 s). All photothermal agents were mixed CNFs at the same ratio and prepared as solutions of the same concentration and finally coated on WYMs with the same process.

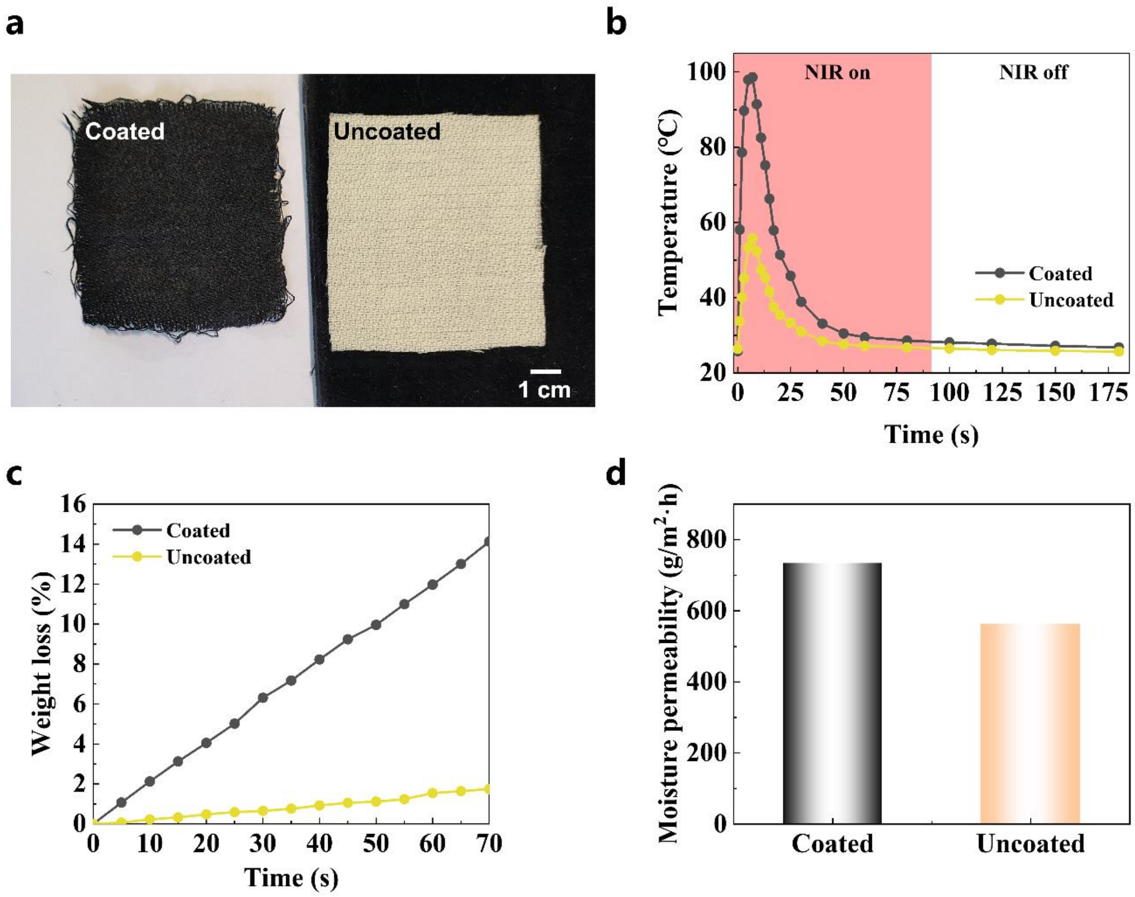

Figure S5. (a) Photographs of the wool fabric coated and uncoated with MXene/CNFs. (b) Temperature profile when NIR light (1.25 W cm^{-2}) was turned on and off for different wool fabrics. (c) Weight loss as a function of time under 700 mW cm^{-2} NIR light (70 s). (d) Moisture permeability of the wool fabric coated and uncoated with MXene/CNFs.

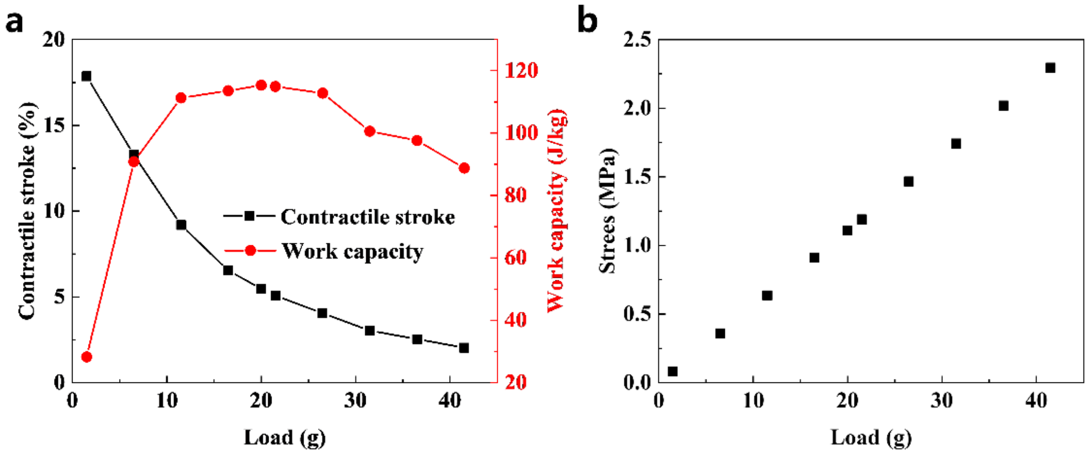

Figure S6. Weight lifting performance of a self-plied MCWYM. (a) Dependence of the contractile stroke and work capacity on the weight of the applied load. (b) Correspondence between the applied stress and mass of the applied load.

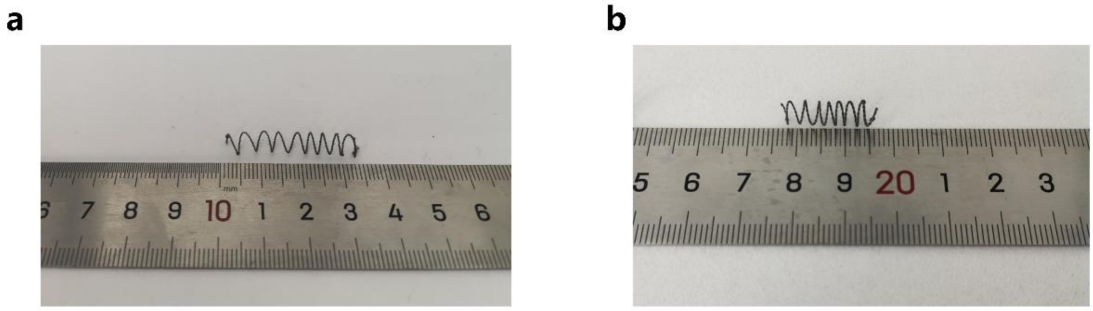

Figure S7. Photos of typical coiled MCWYMs with (a) heterochiral and (b) homochiral coils.

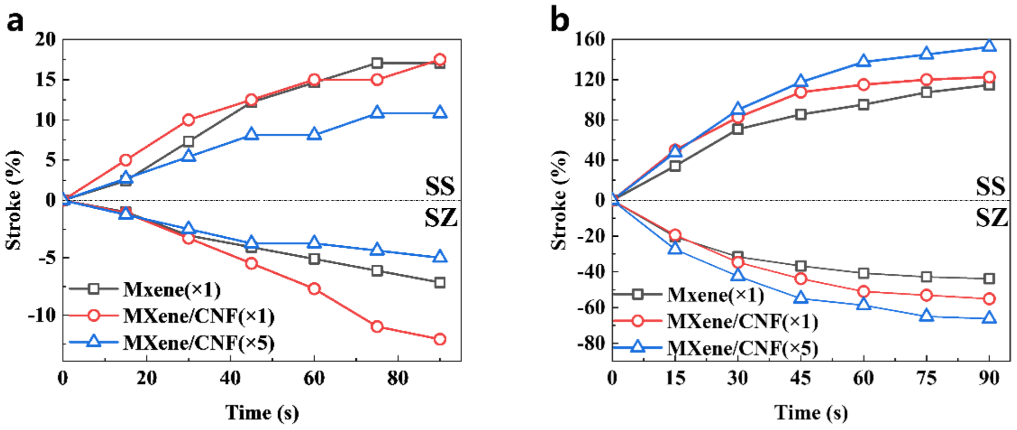

Figure S8. Actuation stroke of different coiled WYMs coated with MXene (1 time), MXene /CNF (1 time), or MXene /CNF (5 times) under a NIR power density of (a) 100 mW cm^{-2} and (b) 700 mW cm^{-2}.

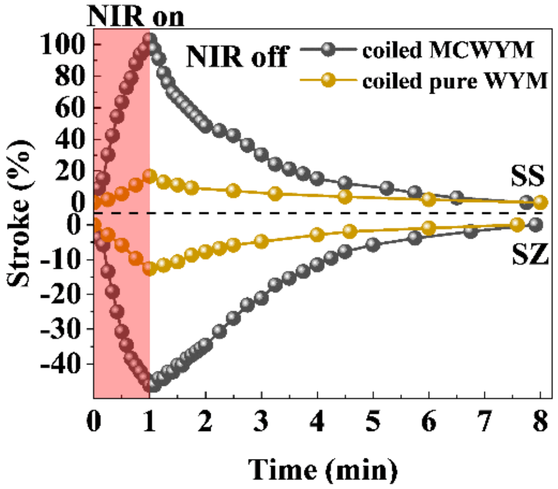

Figure S9. Comparison of photothermal actuation and recovery of coiled MCWYMs and coiled pure WYMs (without MXene/CNF coating) under 60% RH. The NIR light irradiation was 500 mW cm^{-2}.

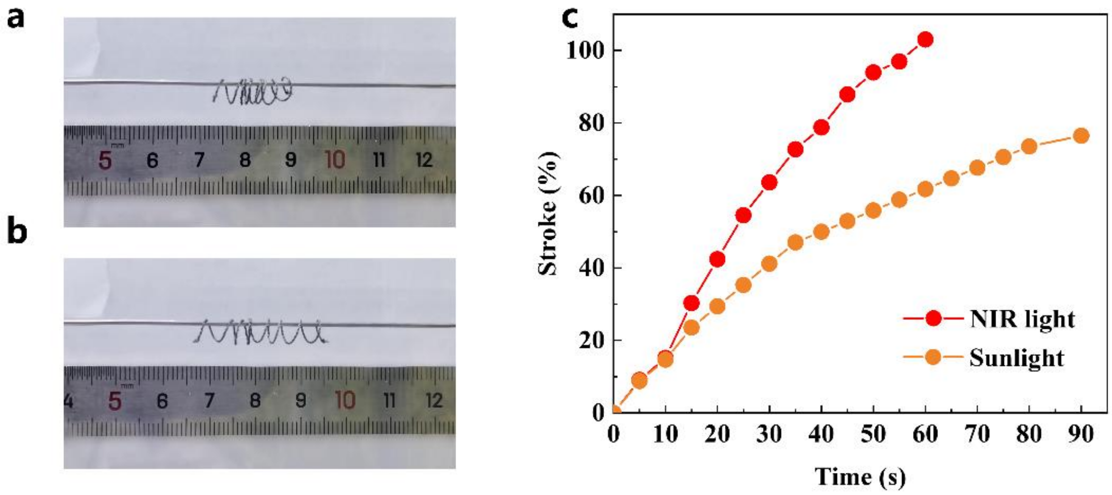

Figure S10. Actuation performance of a homochirally coiled MCWYM under natural sunlight. (a, b) Photographs of the MCWYM (a) before and (b) after being irradiated by natural sunlight for 90 s. (c) Comparison of the actuation strokes under natural sunlight or a NIR light of 500 mW cm^{-2} for different durations.

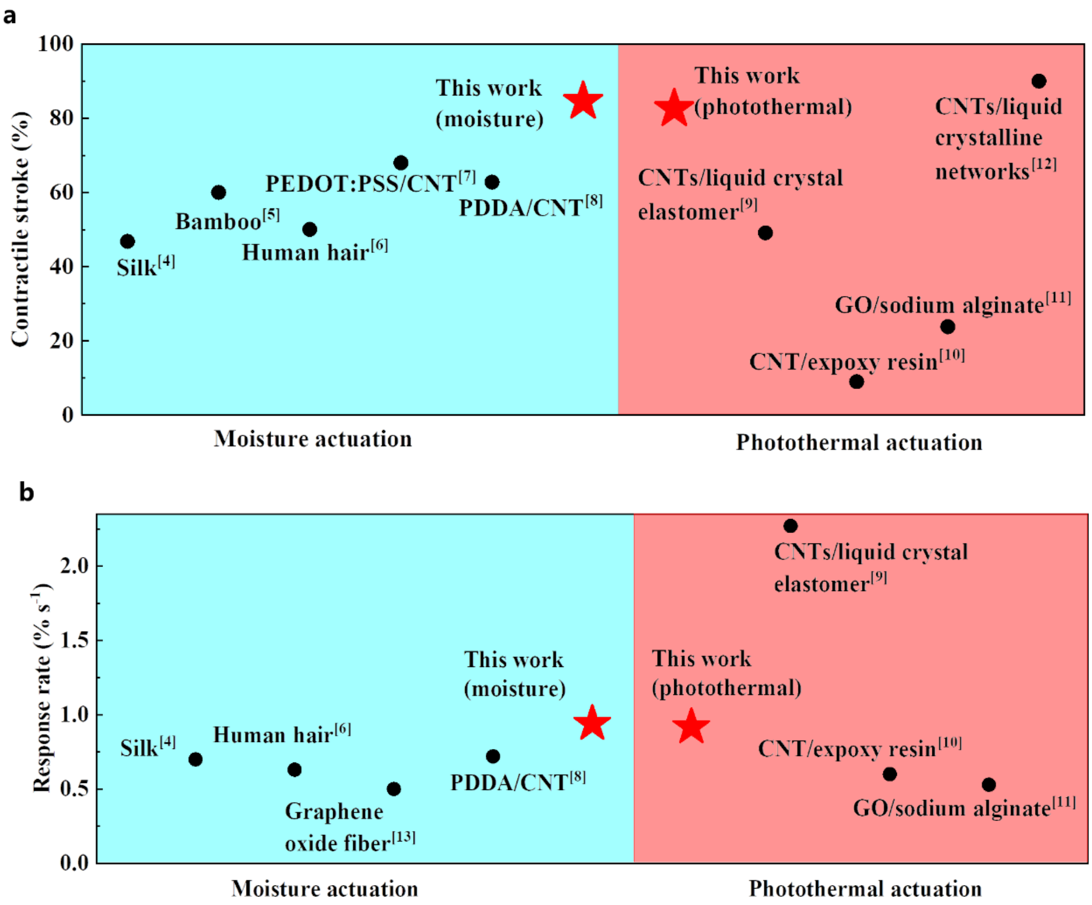

Figure S11. Comparison of the (a) contraction stroke and the (b) response rate of the coiled MCWYM with previously reported moisture-responsive or photothermal fiber-based muscles. The moisture response stroke of the coiled MCWYM was regarded as the photothermal elongation stroke. The reported results of moisture actuation under 90% RH or water fog with similar process parameters (such as spring index, twist density, etc.) were selected.

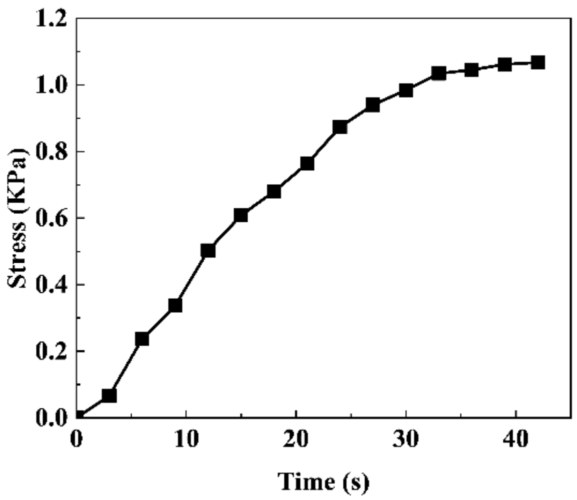

Figure S12. Blocking stress of a homochirally (SS) coiled MCWYM under NIR light (700 W cm^{-2}) irradiation. The blocking stress was calculated from the blocking force generated by the coiled MCWYM.

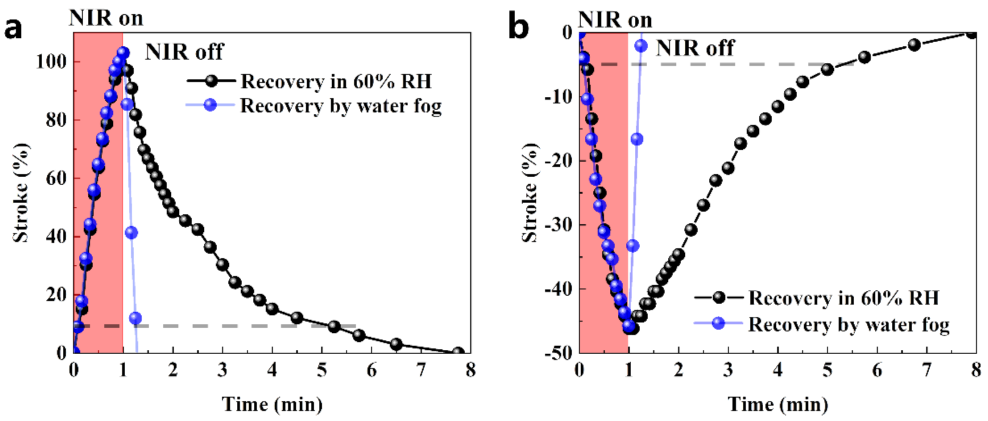

Figure S13. The actuation stroke of coiled MCWYMs during photothermal actuation (all at 60% RH) and the recovery process under 60% RH (black) or water fog (blue). (a) Homochirally coiled (SS) MCWYMs. (b) Heterochirally coiled (SZ) MCWYMs. The NIR light illumination intensity for photothermal actuation was 500 mW cm^{-2}. In the case of recovery by water fog, the MCWYM was actuated under NIR irradiation in 60% RH but exposed to water fog immediately after the NIR light was off.

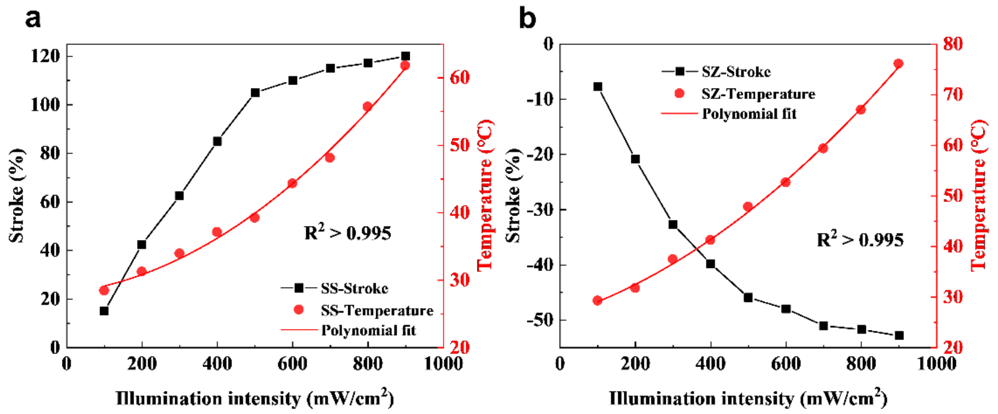

Figure S14. Dependence of actuation stroke and temperature rise of different coiled MCWYMs on the irradiated NIR light power density. (a) A homochirally coiled (SS) MCWYM. (b) A heterochirally coiled (SZ) MCWYM. The temperature data set is fitted with a polynomial curve with $R^2 > 0.995$.

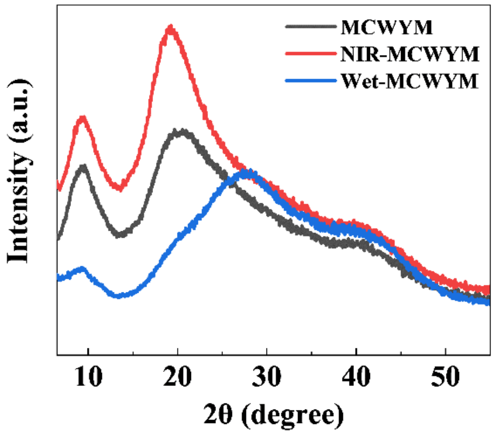

Figure S15. XRD patterns of MCWYM samples under different states. The samples exposed to 700mW cm^{-2} NIR light and water fog for 90 s were named as NIR-MCWYM and Wet-MCWYM, respectively.

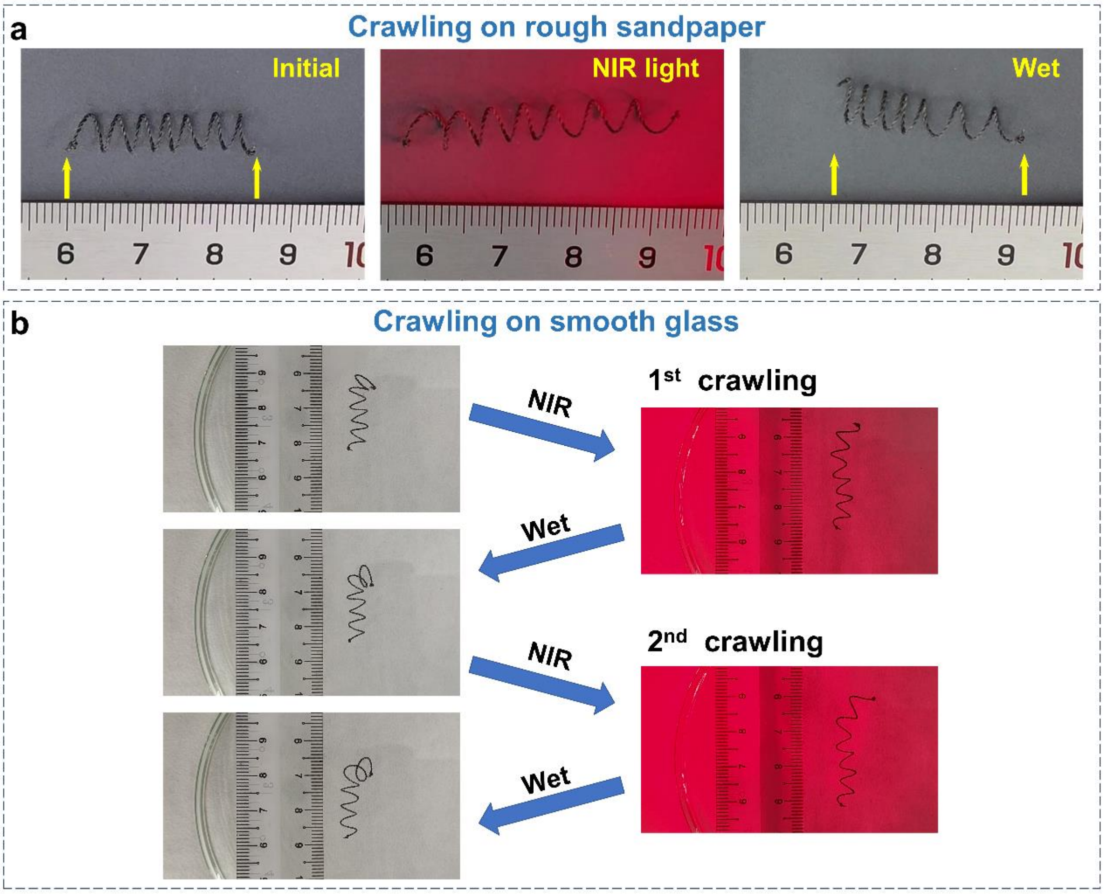

Figure S16. Sequential photos showing the robotic caterpillar crawling progressively on (a) a smooth glass and (b) rough sandpaper, which was alternatively driven by biased moisture exposure and NIR light irradiation.

Supplementary References

[1] M. Alhabeb, K. Maleski, B. Anasori, P. Lelyukh, L. Clark, S. Sin, Y. Gogotsi, *Chemistry of Materials* **2017**, 29, 7633.

[2] G. Cai, J.-H. Ciou, Y. Liu, Y. Jiang, P. S. Lee, *Science Advances* **2019**, 5, eaaw7956.

[3] S. Chen, J.-H. Ciou, F. Yu, J. Chen, J. Lv, P. S. Lee, *Advanced Materials* **2022**, 34, 2200660.

[4] T. Jia, Y. Wang, Y. Dou, Y. Li, M. Jung de Andrade, R. Wang, S. Fang, J. Li, Z. Yu, R. Qiao, *Advanced Functional Materials* **2019**, 29, 1808241.

[5] X. Hu, X. Leng, T. Jia,, Z. Liu, *Chin. Phys. B* **2020**, 29, 118103.

[6] X. Leng, X. Zhou, J. Liu, Y. Xiao, J. Sun, Y. Li, Z. Liu, *Materials Horizons* **2021**, 8, 1538.

[7] X. Gu, Q. Fan, F. Yang, L. Cai, N. Zhang, W. Zhou, W. Zhou, S. Xie, *Nanoscale* **2016**, 8, 17881.

[8] S. H. Kim, C. H. Kwon, K. Park, T. J. Mun, X. Lepró, R. H. Baughman, G. M. Spinks, S. J. Kim, *Scientific Reports* **2016**, 6, 23016.

[9] Y. Yu, L. Li, E. Liu, X. Han, J. Wang, Y.-X. Xie, C. Lu, *Carbon* **2022**, 187, 97.

[10] L. Xu, Q. Peng, X. Zhao, P. Li, J. Xu, X. He, *ACS Applied Materials & Interfaces* **2020**, 12, 40711.

[11] W. Wang, C. Xiang, D. Sun, M. Li, K. Yan, D. Wang, *ACS Applied Materials & Interfaces* **2019**, 11, 21926.

[12] Z.-C. Jiang, Y.-Y. Xiao, R.-D. Cheng, J.-B. Hou, Y. Zhao, *Chemistry of Materials* **2021**, 33, 654.

[13] H. Cheng, J. Liu, Y. Zhao, C. Hu, Z. Zhang, N. Chen, L. Jiang, L. Qu, *Angewandte Chemie International Edition* **2013**, 52, 10482.
